# Public Health Response to the Initiation and Spread of Pandemic COVID-19 in the United States, February 24–April 21, 2020

**DOI:** 10.15585/mmwr.mm6918e2

**Published:** 2020-05-08

**Authors:** Anne Schuchat, CDC COVID-19 Response Team

From January 21 through February 23, 2020, a total of 14 cases of coronavirus disease 2019 (COVID-19) were diagnosed in six U.S. states, including 12 cases in travelers arriving from China and two in household contacts of persons with confirmed infections. An additional 39 cases were identified in persons repatriated from affected areas outside the United States (*1*). Starting in late February, reports of cases with no recent travel to affected areas or links to known cases signaled the initiation of pandemic spread in the United States (*2*). By mid-March, transmission of SARS-CoV-2, the virus that causes COVID-19, had accelerated, with rapidly increasing case counts indicating established transmission in the United States. Ongoing traveler importation of SARS-CoV-2, attendance at professional and social events, introduction into facilities or settings prone to amplification, and challenges in virus detection all contributed to rapid acceleration of transmission during March. Public health responses included intensive efforts to detect cases and trace contacts, and implementation of multiple community mitigation strategies. Because most of the population remains susceptible to infection, recognition of factors associated with amplified spread during the early acceleration period will help inform future decisions as locations in the United States scale back some components of mitigation and strengthen systems to detect a potential transmission resurgence. U.S. circulation of SARS-CoV-2 continues, and sustained efforts will be needed to prevent future spread within the United States.

The first cases of COVID-19 in the United States occurred in January and February 2020 in travelers from China’s Hubei Province, where the virus was first recognized, and their household contacts (*1*). Beginning in late February, cases with no history of international travel and no contact with infected persons were recognized (*1*). By mid-March, transmission had become widespread, and by April 21, a total of 793,669 confirmed COVID-19 cases had been reported in the United States, the majority resulting from widespread community transmission (Figure 1). Factors that contributed to the acceleration of dissemination in March included 1) continued importation of the virus by travelers infected elsewhere (e.g., on cruise ships or in countries experiencing outbreaks); 2) attendance at professional and social events, resulting in amplification in the host locations and multistate spread; 3) introduction of the virus into facilities or settings prone to amplification (e.g., long-term care facilities and high-density urban areas) with the potential for seeding the broader community; and 4) challenges in virus detection, including limited testing, emergence during the peak months of influenza circulation and influenza and pneumonia hospitalizations, and other cryptic transmission including from persons who were asymptomatic or presymptomatic. During March 2020, national, state, and local public health responses also intensified and adapted, augmenting case detection, contact tracing, and quarantine with targeted layered community mitigation measures. Because SARS-CoV-2, the virus that causes COVID-19, remains in circulation and a large proportion of the population remains susceptible, the potential for future acceleration remains.

**FIGURE 1:**
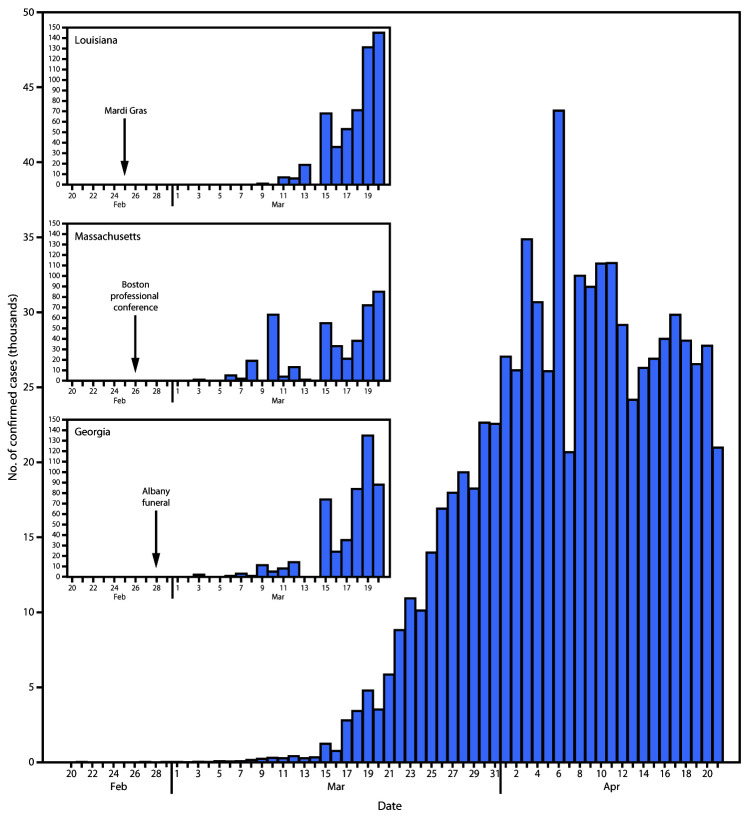
Number of confirmed COVID-19 cases, by date of report, in the United States during February 20–April 21, 2020,* with initiation and early acceleration periods highlighted in Louisiana, Massachusetts, and Georgia **Abbreviation: **COVID-19 = coronavirus disease 2019. *Cumulative case count was 13 before February 20, 2020.

## Travel and COVID-19 Spread

**FIGURE 2:**
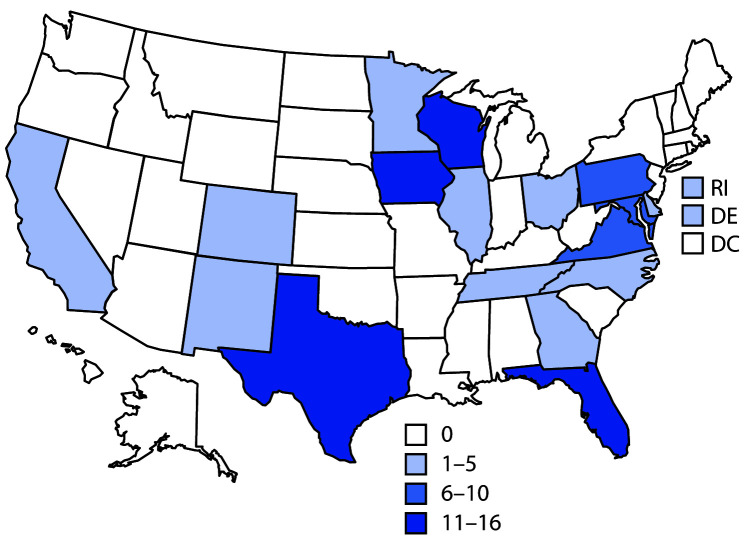
Number of confirmed COVID-19 cases (N = 101) linked to nine Nile River cruises held during February 11–March 5, 2020, by patient state of residence — 18 states **Abbreviations: **COVID-19 = coronavirus disease 2019; DC = District of Columbia; DE = Delaware; RI = Rhode Island.

Continued introductions of SARS-CoV-2 from outside the United States contributed to the initiation and acceleration of domestic COVID-19 cases in March. After Chinese authorities halted travel from Wuhan and other cities in Hubei Province on January 23, followed by U.S. restrictions on non-U.S. travelers from China issued on January 31 (effective February 2), air passenger journeys from China decreased 86%, from 505,560 in January to 70,072 in February. However, during February, 139,305 travelers arrived from Italy and 1.74 million from all Schengen countries,* where the outbreak was spreading widely and rapidly. Travelers from Italy and all Schengen countries decreased 74% to 35,877 and 50% to 862,432, respectively, in March.^†^ Genomic analysis of outbreak strains suggested an introduction from China to the state of Washington around February 1.^§^ However, examination of strains collected from northern California during early February to mid-March indicated multiple introductions resulting from international travel (from China and Europe) as well as from interstate travel.^¶^ Sequencing of strains collected in the New York metropolitan area in March also suggested origins in Europe and other U.S. regions.** Returning cruise ship travelers also contributed to amplification during this time (*3*). Persons from many countries are in close contact on cruises, and crew members continue to work on ships for multiple voyages. As a result, passengers returning from cruises contributed to the early acceleration phase. For example, 101 persons who had been on nine separate Nile River cruises during February 11–March 5 returned to 18 states and had a positive test result for SARS-CoV-2, nearly doubling the total number of known COVID-19 cases in the United States at that time (Figure 2).

Public health steps to mitigate continued importations of the virus included travel restrictions for non-U.S. citizens or permanent residents arriving from China beginning in early February and later expanded to include other countries with widespread sustained transmission (Table). Travel health notices were issued for countries with known outbreaks as the pandemic evolved, and ultimately warnings were issued to avoid nonessential international travel as well as all cruise ship travel (*1*,*4*). Quarantine measures were implemented for arriving international travelers with known exposure to locations and settings of concern, such as Hubei Province and the *Diamond Princess* cruise ship docked off the coast of Yokohama, Japan. Screening and public health risk assessment of travelers in selected U.S. airports, initiated on January 17, were also expanded. As of April 21, 2020, CDC staff members and U.S. Customs and Border Protection officers had screened approximately 268,000 returning travelers, among whom testing confirmed 14 COVID-19 cases. State and local health departments were advised to supervise self-monitoring of travelers who had been directed to stay home after returning from countries with widespread sustained transmission. On March 14, 2020, the CDC Director issued a No Sail Order for cruise ships, suspending operation in U.S. waters; the order was renewed April 9, effective April 15.

**TABLE:** Factors contributing to COVID-19 acceleration and corresponding public health actions — United States, January–April 2020

Factor contributing to acceleration	Examples	Public health actions
Continued travel-associated importations of the virus	Travelers arriving from countries or cruise ships with ongoing transmission	Travel health notices, traveler screening (including risk assessment, public health management and monitoring), travel restrictions, federal isolation and quarantine orders, educating travelers and clinicians regarding symptoms and evaluation
Large gatherings	Social, cultural, and professional gatherings where persons convene and then disperse over broad areas	Restricting mass gatherings; global travel restrictions and domestic travel recommendations, recommending transition to virtual events
Introductions into high-risk workplaces/settings	Long-term care facilities, hospitals, correctional facilities, and homeless shelters	Restricting visitor access, establishing cohort units or facilities for residential settings, vigorous contact tracing around persons with confirmed cases, increased infection control, environmental surface cleaning, use of recommended personal protective equipment
Crowding and high population density	Densely populated areas, crowded workplaces, schools, and public spaces	Stay-at-home orders, recommendations for hand washing and social distancing, cloth face covering guidance, school dismissals, extended telework, environmental surface cleaning
Cryptic transmission	Presymptomatic or asymptomatic spread, limited testing, co-occurrence with circulation of other respiratory viruses	Increased testing, COVID-19–specific surveillance, cloth face covering guidance, aggressive contact tracing accompanied by quarantine and/or testing of asymptomatic contacts, stay-at-home orders

**Abbreviation: **COVID-19 = coronavirus disease 2019.

## Events and Gatherings

Various gatherings of persons from different locations, followed by return to their home communities, played a notable role in the early U.S. spread of COVID-19. During February 2020, the number of confirmed cases originating in the United States was low and appeared contained; thus, federal and local jurisdictions did not recommend restrictions on gatherings. However, during the last week of February, several large events led to further spread of the disease. These included Mardi Gras celebrations in Louisiana with more than 1 million attendees, an international professional conference held in Boston, Massachusetts, with approximately 175 attendees, and a funeral in Albany, Georgia, with more than 100 attendees (Figure 1). In the weeks after these events, amplifications in the host locations contributed to increasing U.S. case counts (*5*). Dougherty County, Georgia, a small rural county that includes Albany, had one of the highest cumulative incidences of COVID-19 (1,630/100,000 population) in the country. The substantial transmissibility of the virus and severity of COVID-19 triggered a series of recommendations, beginning in mid-March, to limit mass gatherings and travel (Table).

## Workplaces and Settings Contributing to Accelerated Spread

Skilled nursing and long-term care facilities (*6*) and hospitals (*7*) are settings in which persons at higher risk for severe COVID-19 illness are in close contact with staff members, many of whom work at multiple facilities. Other workplaces also facilitated amplification of virus transmission, including critical infrastructure sectors, such as multiple meat packing facilities in rural areas. Clusters of cases related to religious service attendance have been reported within the United States and worldwide (*8*). Congregate, high-density settings also might contribute to the spread of COVID-19 (*9*). For example, population density might account for the very high numbers of COVID-19 cases in the New York metropolitan area (Box). Public health actions aimed at reducing COVID-19 spread in high-risk settings have focused on infection control measures, including identifying and isolating ill persons, cleaning and disinfection, restricting visitors, physical distancing through shift work, and appropriate use of personal protective equipment (Table). To protect health care capacity and slow community spread of COVID-19, local, state, and federal authorities issued stay-at-home orders, and closed schools and nonessential workplaces. On April 3, CDC issued guidance for use of cloth face coverings in public areas to reduce spread, based on increasing evidence of transmission in the absence of symptoms.^††^

BOX **Critical factors contributing to COVID-19 spread in New York**

Multiple interrelated factors that complicated identification and isolation of cases and tracing of contacts contributed to the COVID-19 outbreak in New York.

**Population density**

- New York City’s boroughs represent the top four population-dense U.S. counties.
- Reliance on mass transit (subways, buses, and ferries) results in frequent, prolonged close contact.
- High prevalence of apartment living contributed to household spread.

**Domestic and global destination**

- Three major airports serve as domestic and global hubs, serving >1 million air passengers per week.
- Approximately 1.6 million persons commute into Manhattan daily during the work week, primarily using mass transit.

**Large number of crowded settings housing vulnerable populations**

- **Long-term care facilities, skilled nursing facilities**: At least 80 facilities in the state have reported five or more cases as of April 21; initial infections were noted in early March.
- **Correctional institutions**: As of April 21, incidence in Department of Corrections and Community Supervision facilities was approximately seven times that in the state overall.
- **Homeless shelters**: As of the week of April 21, approximately 600 cases were confirmed among shelter residents and other persons experiencing homelessness.

**Large gatherings**

- Initial cases in Westchester County were associated with attendance at large gatherings in late February.
- All types of large work and social gatherings accelerated transmission across jurisdictional boundaries.

## Cryptic Transmission

Unrecognized transmission played a key role in the initiation and acceleration phases of the U.S. outbreak. Cases were not detected during this time for various reasons. First, introduction of the virus into the United States occurred during the annual influenza season. Although syndromic surveillance systems tracked respiratory illness in outpatient settings and emergency departments in many U.S. jurisdictions, including areas where early COVID-19 clusters were detected, such as Seattle, Washington, none of these systems detected unusual trends during the early part of the acceleration period because of the preponderance of seasonal influenza illness. After the first community case in Santa Clara, California, was confirmed on February 27, the county conducted COVID-19 surveillance with polymerase chain reaction–based virus testing during March 5–14 at four urgent care centers. Influenza accounted for 23% of respiratory illnesses; among those who had a negative test result for influenza, 11% had a positive test result for SARS-CoV-2, representing approximately 8% of patients with respiratory symptoms (*10*). Seroprevalence data from Seattle during March 2020, a period when transmission of the virus was rapidly accelerating, suggested that there were limited undetected infections in healthy adults without respiratory illness (1 of 221 remnant clinical sera representing a convenience sample tested seropositive [Helen Chu, University of Washington School of Public Health, personal communication, April 2020]); at the population level, this still translates into substantial numbers of unrecognized community infections. No samples from 59 children with acute respiratory infections during January–March were seropositive (Janet Englund, Seattle Children’s Hospital and University of Washington, personal communication, April 2020). Because the incidence of SARS-CoV-2 infections was still relatively low during the initiation and early acceleration periods, as evidenced by seroprevalence data, widespread testing would have been needed to detect all cases. The contribution of spread from persons without symptoms also complicated detection and containment (*11*). Public health actions included expanded surveillance and testing capacity and community measures, such as enhanced teleworking and stay-at-home orders, school closures, social distancing, and use of cloth face coverings (Table).

## Discussion

The acceleration phase of a pandemic is complex and requires a multifaceted and rapidly adapting public health response. During a 3-week period in late February to early March, the number of U.S. COVID-19 cases increased more than 1,000-fold. Various community mitigation interventions were implemented with the aim of reducing further spread and controlling the impact on health care capacity. Recognition of factors associated with amplified spread during this early acceleration period will help inform future decisions as locations in the United States scale back some components of mitigation and strengthen systems to detect transmission resurgence.

The findings in this report are subject to at least five limitations. First, the various factors facilitating viral spread described in this report occurred simultaneously; therefore, it is not possible to quantify the relative contribution of each to the outbreak trajectory in the United States. Second, the examples of factors contributing to amplification are illustrative and not meant to be comprehensive. Third, because the mitigation strategies highlighted here were implemented concurrently, the ability to estimate the relative impact of each intervention is limited. Fourth, the epidemic curve presented was likely affected by limited testing, particularly in the early phases of the outbreak. Finally, the case counts presented are an underestimate of the actual number of COVID-19 cases in the United States.

As the pandemic evolves, control efforts must be continuously refined. Certain interventions that were critical in the early stages, such as quarantine and airport screening, might have less impact when transmission is widespread in the community. However, many elements of the mitigation strategies used during the acceleration phase will still be needed in later stages of the outbreak. Preliminary results from serologic surveys suggest that even in the U.S. regions with the largest numbers of recognized cases, most persons have not been infected and remain susceptible.^§§^^,^^¶¶^ Therefore, sustained and concerted efforts will be needed to prevent future spread of SARS-CoV-2 within the United States.

**Summary**

**What is already known about this topic?**

The first confirmed coronavirus disease 2019 (COVID-19) case in the United States was reported on January 21, 2020. The outbreak appeared contained through February, and then accelerated rapidly.

**What is added by this report?**

Various factors contributed to accelerated spread during February–March 2020, including continued travel-associated importations, large gatherings, introductions into high-risk workplaces and densely populated areas, and cryptic transmission resulting from limited testing and asymptomatic and presymptomatic spread. Targeted and communitywide mitigation efforts were needed to slow transmission.

**What are the implications for public health practice?**

Factors that amplified the March acceleration and associated mitigation strategies that were implemented can inform public health decisions as the United States prepares for potential re-emergences.

## Footnotes

* Includes Austria, Belgium, Czech Republic, Denmark, Estonia, Finland, France, Germany, Greece, Hungary, Iceland, Italy, Latvia, Liechtenstein, Lithuania, Luxembourg, Malta, Netherlands, Norway, Poland, Portugal, Slovakia, Slovenia, Spain, Sweden, and Switzerland. Travel within the Schengen Area is permitted without border controls.

† Air travel data provided by U.S. Customs and Border Protection’s Office of Planning, Program Analysis, and Evaluation (PPAE).

§ https://www.medrxiv.org/content/10.1101/2020.04.02.20051417v2.

¶ https://www.medrxiv.org/content/10.1101/2020.03.27.20044925v1.

** https://www.medrxiv.org/content/10.1101/2020.04.08.20056929v2.

†† https://www.cdc.gov/coronavirus/2019-ncov/prevent-getting-sick/cloth-face-cover.html.

§§ https://www.governor.ny.gov/news/video-audio-photos-rush-transcript-amid-ongoing-covid-19-pandemic-governor-cuomo-announces-12.

¶¶ https://www.medrxiv.org/content/10.1101/2020.04.14.20062463v1.
